# Hydrogel-Inducing Graphene-Oxide-Derived Core–Shell Fiber Composite for Antibacterial Wound Dressing

**DOI:** 10.3390/ijms24076255

**Published:** 2023-03-26

**Authors:** Yuliya Kan, Julia V. Bondareva, Eugene S. Statnik, Elizaveta V. Koudan, Evgeniy V. Ippolitov, Mikhail S. Podporin, Polina A. Kovaleva, Roman R. Kapaev, Alexandra M. Gordeeva, Julijana Cvjetinovic, Dmitry A. Gorin, Stanislav A. Evlashin, Alexey I. Salimon, Fedor S. Senatov, Alexander M. Korsunsky

**Affiliations:** 1 Skolkovo Institute of Science and Technology, Bolshoy Boulevard 30, bld. 1, 121205 Moscow, Russia; 2 Center for Biomedical Engineering, National University of Science and Technology ‘MISIS’, Leninskiy pr. 4, 119049 Moscow, Russia; 3 Department of Microbiology, Virology, Immunology, A.I. Yevdokimov Moscow State University of Medicine and Dentistry, Delegatskaya St. 20, 127473 Moscow, Russia; 4 Department of Chemistry and BINA—BIU Center for Nanotechnology and Advanced Materials, Bar-Ilan University, Ramat-Gan 5290002, Israel; 5 Multi-Beam Laboratory for Engineering Microscopy, Department of Engineering Science, University of Oxford, Parks Road, Oxford OX1 3PJ, UK

**Keywords:** nanofiber, graphene oxide, silica, crosslinking, wound dressing

## Abstract

The study reveals the polymer–crosslinker interactions and functionality of hydrophilic nanofibers for antibacterial wound coatings. Coaxial electrospinning leverages a drug encapsulation protocol for a core–shell fiber composite with a core derived from polyvinyl alcohol and polyethylene glycol with amorphous silica (PVA-PEG-SiO_2_), and a shell originating from polyvinyl alcohol and graphene oxide (PVA-GO). Crosslinking with GO and SiO_2_ initiates the hydrogel transition for the fiber composite upon contact with moisture, which aims to optimize the drug release. The effect of hydrogel-inducing additives on the drug kinetics is evaluated in the case of chlorhexidine digluconate (CHX) encapsulation in the core of core–shell fiber composite PVA-PEG-SiO_2_-1x-CHX@PVA-GO. The release rate is assessed with the zero, first-order, Higuchi, and Korsmeyer–Peppas kinetic models, where the inclusion of crosslinking silica provides a longer degradation and release rate. CHX medicated core–shell composite provides sustainable antibacterial activity against *Staphylococcus aureus*.

## 1. Introduction

Varied configurations and origins of wounds, and bacterial invasions, require specific remedies. One of the healing issues is dense biofilm formation as a result of bacterial inflammation. *Staphylococcus aureus*, being a widely distributed Gram-positive bacterium, is mainly responsible for skin and soft tissue infections. Chronic diabetic ulcers, complex burns, and periprosthetic infections are sensitive to the bacterial activity that is attributed to the hard-to-treat biofilm [1]. The hyperglycemic environment facilitates the bacteria’s colonization and damage of the vascular network, preventing the rehabilitation of diabetic patients [2,3]. Antibiotic therapy carries an increased risk of side effects such as intractable infections [4]. For periprosthetic infections, it is challenging to remove a biofilm from the infected implant.

Hence, the issues of long-healing and chronic wounds relate to bacterial and surgical infections, resulting in a painful and protracted treatment. The development of a bioresorbable wound healing composite aims to reduce surgical invasion. The hydrophilic and gel composites are considered in terms of retaining a sufficient level of moisture for cell migration. One of the most promising directions is the fiber–hydrogel suggested in the studies [5,6,7,8,9,10,11]. Additionally, the wound dressing should display antibacterial activity to support the regeneration of tissues.

The abundance of spinnable polymers enables the development of fiber composites. Hydrophilic polymers such as polyvinyl alcohol (PVA), polyethylene glycol (PEG), and polyvinyl pyrrolidone (PVP) are considered due to the moisture regulation and non-toxicity of the decomposition products [12,13,14,15]. The PVA-PVP hydrogel composite displays the high potential of hydrophilic polymers to promote healing by sufficient cell migration [14]. The regulation of exudates is an important condition for tissue regeneration. The optimal level of moisture supports the activity of epithelial cells in the wound bed, which plays a major role in healing. PVA hydrogels are introduced to solve the hydration issue. The functionality of hydrogels could be enhanced with Ag^+^ ions to prevent the bacterial contamination of wounds and medicals to promote angiogenesis [2,16].

PVA-derived fibers are considered for medicated coatings. Primarily, the fibers could serve as drug carriers for antibiotic, antiviral, and vascular growth agents [17,18]. For instance, a PVA composite loaded with erythromycin showed antibacterial effects against *Staphylococcus aureus* and *Escherichia coli* [19]. In the relevant study [4], medicated polycaprolactone-derived fibers were suggested against a periprosthetic infection. The majority of infections are caused by S. aureus bacteria that colonize the implant with a hard-to-remove biofilm, inducing severe inflammation.

Due to the good miscibility of PVA with hydrogel-inducing, functionalized additives, there are several directions for composites. The crosslinking helps to achieve the optimal rate of dissolution, and mechanical properties are enhanced by modifications of the polymer network via hydrogen and covalent bonds [20].

Hydroxyl groups of PVA interact with the nanosized components such as cellulose via intermolecular hydrogen bonding [21,22]. Hydrogen bonding was achieved in a study of a PVA and nanocellulose-based composite [22], where the involvement of hydroxyl groups was shown spectroscopically by the reduced OH vibrations for crosslinked samples. A hydrogen-crosslinked PVA–chitosan fiber composite was suggested for ocular treatment against bacterial conjunctivitis [10]. Chitosan is a hydrogel-inducing additive that accelerates the gelation process. PVA–chitosan nanofibers display biocompatibility, with cell viability > 70% for L929 mouse fibroblasts.

Covalent crosslinking helps to tune the physical properties of polyethylene-oxide-based composites, offering self-healing and mechanical properties, as the authors suggested in [23]. Notably, hydrophilic and hydrophobic polymers such as polystyrene and poly(4-vinyl pyridine) were covalently crosslinked to build a hydrogel composite [24]. Yield dynamic covalent bonds were triggered with UV, initiating the self-healing of the produced hydrogel. The crosslinking features of hydrophilic polymer fibers are exploited in the hydrogels applied for antibacterial and hemostatic wound dressings using non-toxic crosslinking agents [8,25].

Chlorhexidine digluconate (CHX) is a non-antibiotic, antiseptic drug in wound dressing studies that is water-dissolved in the spinning solution and monitored with spectroscopic techniques. It is well adapted to treat wound lesions due to its bactericidal and bacteriostatic properties. Hydrophilic drugs such as CHX are effectively encapsulated within polymer fibers including PVA [26,27]. Actual referenced studies claim the feasibility of CHX to treat burns and in the healing of human gingival tissues [28,29].

In this study, the encapsulation of drugs coupled with crosslinking aims to alter the degradation of hydrophilic polymers and drug kinetics by initiating the hydrogel transition for complex wound treatment. The investigation of the effect of modifying additives, such as graphene oxide (GO) and SiO_2_, complements our previous work of core–shell fibers PVA-PEG-SiO_2_@PVA-GO for biomedical applications [30]. Hence, we demonstrate the full characterization of the produced core–shell composite, including drug kinetics and the antibacterial response to Gram-positive bacteria. The suggested biocompatible solutions for CHX-medicated fibers would help to minimize surgical interventions and postoperative pain in patients with chronic wounds.

## 2. Results and Discussion

### 2.1. Analysis of Core–Shell Fiber PVA-PEG-SiO_2_@PVA-GO

#### 2.1.1. Fiber Morphology

**Figure 1:**
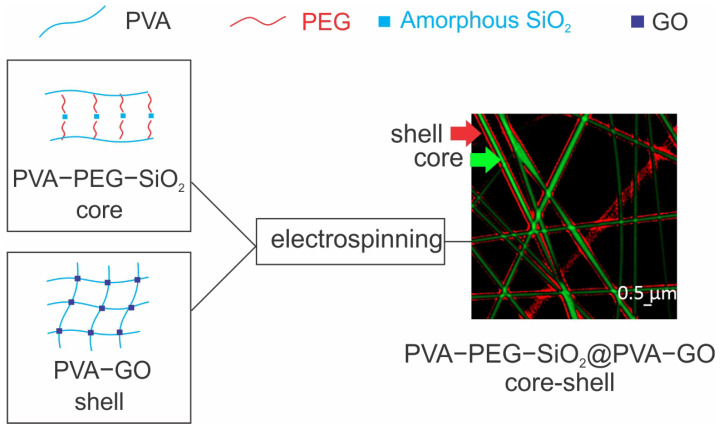
Schematic of fabrication of core–shell fiber composite PVA-PEG-SiO_2_@PVA-GO [30].

The design of the electrospun fiber composite is demonstrated in Figure 1 and a detailed observation of the fiber morphology was presented in our previous work [30]. The core–shell structure was distinguished by confocal laser microscopy with separate fluorescent labeling of the core (Carboxyfluorescein) and shell (Rhodamine B), respectively.

**Figure 2:**
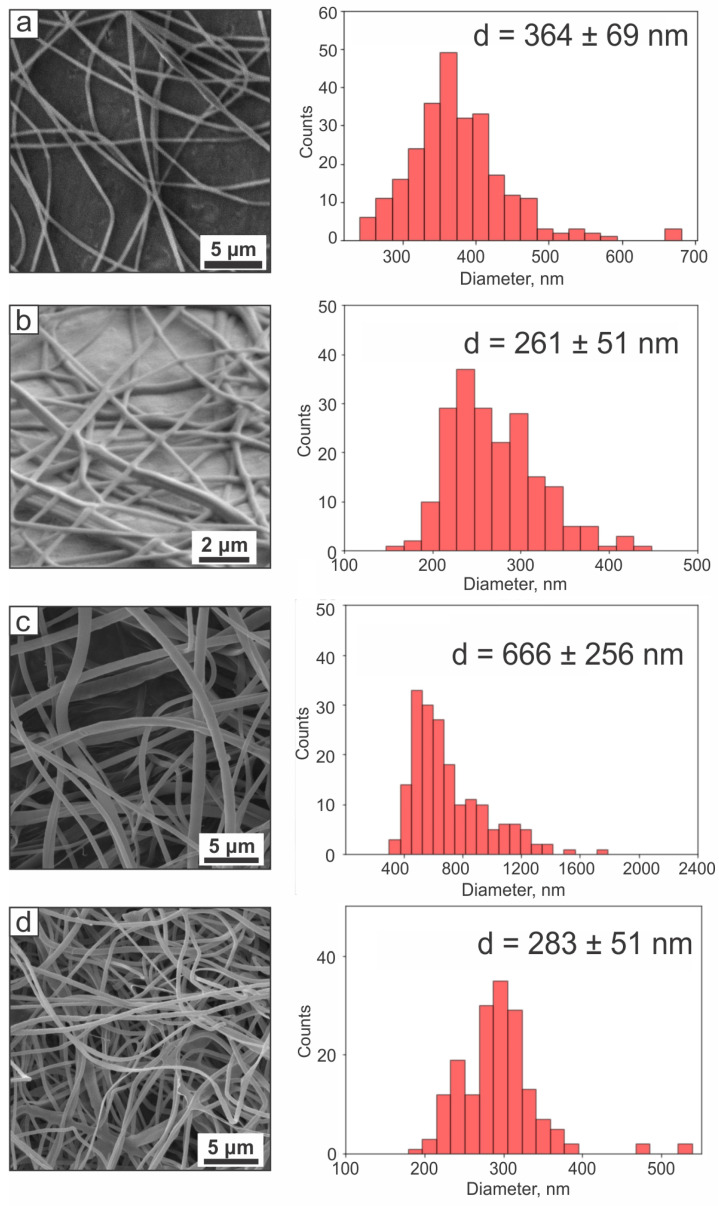
SEM images of fibers with diameter distribution of (**a**) core PVA-PEG-SiO_2_; (**b**) core–shell PVA-PEG-SiO_2_@PVA-GO; (**c**) core PVA-PEG-SiO_2_-1x-CHX; (**d**) core–shell PVA-PEG-SiO_2_-1x-CHX@PVA-GO.

Based on the cumulative characterization retrieved from [30], the PVA-PEG-SiO_2_ core and core–shell PVA-PEG-SiO_2_@PVA-GO fibers have a mean diameter of 364 ± 69 nm and 261 ± 51 nm, respectively, as presented in Figure 2a,b.

For the CHX encapsulation protocol, the drug–polymer interactions and the fiber morphology resulted in the increased fiber diameter of the medicated core and core–shell (Figure 2c,d). The positively charged molecule of CHX facilitates an instant interaction with the negatively charged cell membranes and plays an important role in the produced fiber’s dimensions [31]. Silica and PEG potentially affect the charge density of electrospun solutions, which directly affects the fiber diameter. In the relevant research [32], PEG showed lower electrical conductivity with a subsequently higher fiber diameter. Figure 2 demonstrates that the cationic nature of the encapsulated drug resulted in a larger fiber diameter compared with the ‘empty’ core PVA-PEG-SiO_2_ and core–shell PVA-PEG-SiO_2_@PVA-GO. This drug–polymer interaction requires further investigations as the next direction in this study.

#### 2.1.2. Raman Spectroscopy

**Figure 3:**
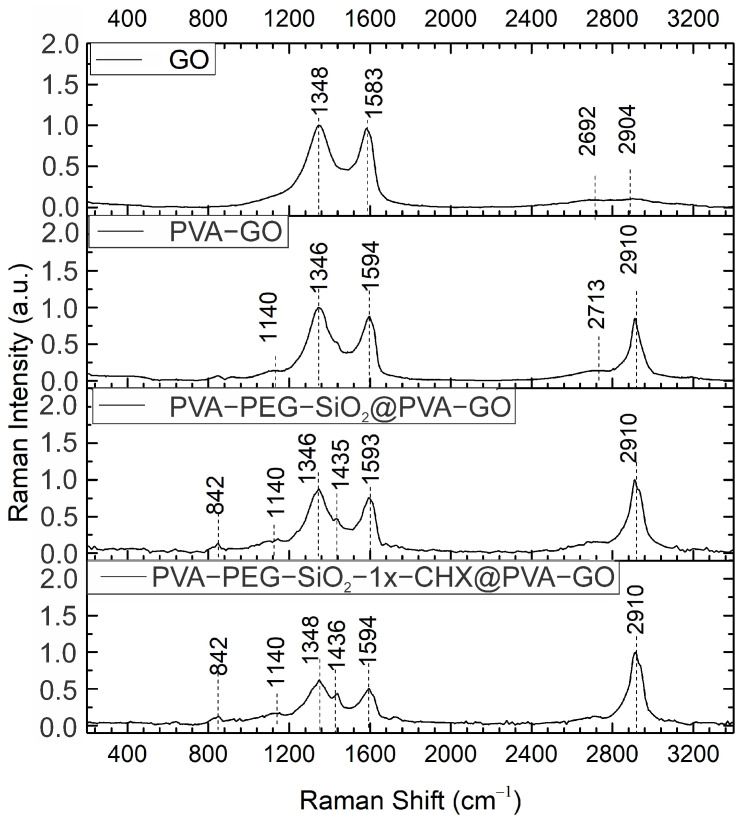
Raman spectra of PVA-based and GO-derived fiber mats in the range of 200–3400 cm^−1^.

In Figure 3, the spectra of PVA-GO present the most prominent peak of PVA occurring at ca. 2910 cm^−1^ and 1435 cm^−1^ due to the C–H bond stretching and symmetric bending vibrations of CH_2_ [33]. The broad intensity at the regions of 1140 cm^−1^ is related to the stretching C–O and C–C bonds within the structure of PVA. The line at 842 cm^−1^ corresponds to C–C stretching bonds and CH_2_ rocking bonds of the polymer part [34].

The PVA-GO shell composite shows the peaks of D and G occurring at ca. 1348 cm^−1^ and a shift from 1583 cm^−1^ to 1594 cm^−1^, respectively. The peaks at 2692 cm^−1^ and 2904 cm^−1^ relate to the 2D band reported from the studies of GO [35]. For PVA-GO, a slight shift in the 2D band is observed at ca. 2700 cm^−1^. The presence of these peaks in the Raman spectra of PVA-GO composites indicates the distribution of the GO flakes in the PVA-GO fibers.

Defining the GO structure, the ratio of ID/IG = 1.03 indicates the high reduction degree of GO. Reduced graphene oxide (rGO) differs due to its higher conductivity and sp^2^ cluster organization, which is important to consider for the electrospinning process [36,37]. The ratios of ID/IG = 1.07 for PVA-GO and ID/IG = 1.14 for core–shell fibers were compared with ID/IG = 1.03 for pristine GO, where the higher level of interaction formed in the electrospun fibers caused a gradual increase in ID/IG [38]. This highlights the next research question regarding how the crosslinking protocol of the spinning solution with GO could alter the conductivity of the spinning solutions and yield fibers. The fabrication of a conductive crosslinked polymer composite opens up broad study opportunities for hydrogel technology, including for the design of drug delivery platforms and wearable sensors [39,40].

#### 2.1.3. Fourier Transform Infrared (FTIR) Spectroscopy

**Figure 4:**
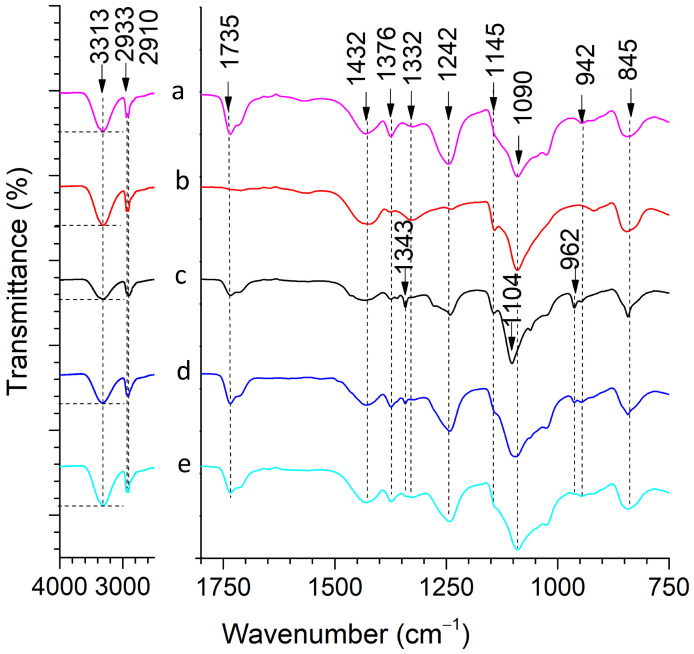
FTIR spectra of (a) PVA 10 wt.% fiber; (b) shell PVA-GO; (c) core PVA-PEG-SiO_2_; (d) core–shell PVA-PEG-SiO_2_@PVA-GO; (e) PVA-PEG-SiO_2_-1x-CHX@PVA-GO.

FTIR spectra of samples are presented in Figure 4. According to the studies, PVA is characterized by the presence of regions for O–H, CH_2_ stretching, CH symmetric bending, CH–OH bending, CH wagging, CH_2_ rocking, and C–C stretching bonds, as listed in Table 1) [41,42,43]. Mainly, the peak located at ca. 1090 cm^−1^ is attributed to the C–O stretching bond of acetyl groups that determine a partially hydrolyzed PVA [44,45]; the intensity of C–O and C–C stretching bonds at 1145 cm^−1^ indicates the degree of crystallinity of PVA [46].

GO has characteristic peaks at 3313 cm^−1^ and 1730 cm^−1^ assigned to the stretching O–H and C=O of carboxyl groups [49,50]. Successful integration of GO flakes within the PVA-GO-derived shell is indicated by the increasing shoulder at 1145 cm^−1^, indicating the formation of a new bond. Meanwhile, the reduced intensity of C=O assigned to carboxyl groups of GO (1730 cm^−1^) indicates their involvement in the hydrogen bonds’ crosslinking.

The spectrum of the core PVA-PEG-SiO_2_ fiber has an increased shoulder (ca. 1145 cm^−1^), defining the silica–polymer interaction in a composite [51]. This bridging effect of silica particles is considered in the literature as the crosslinking approach to establish Si–O–PVA–O–Si interactions. The reduced intensity of OH vibration bands of PVA (3313 cm^−1^) evidences its active role in the building of the composite. There is a visible shift in the peak from 1090 to 1104 cm^−1^, assigned to the asymmetric stretching bonds Si–O at 1104 cm^−1^, which is mentioned in the literature [52]. Following the referenced studies on silica, the characteristic peaks occurred at 1020–1110 cm^−1^ and defined the asymmetric stretching vibration of Si–O–Si bonds, and the peak at 962 cm^−1^ proves the formation of the asymmetric bending and stretching vibration of silanol groups Si–OH [53]. The presence of silanol groups serves to alter the water solubility and drug release behavior of PVA-PEG-SiO_2_ core via stronger intermolecular interactions.

As a result, the FTIR measurements indicate the crosslinking of the core and shell fibers. The FTIR spectra of core–shell fiber PVA-PEG-SiO_2_@PVA-GO and PVA-PEG-SiO_2_-1x-CHX@PVA-GO loaded with CHX were compared to find the crystalline peaks attributed to the CHX; however, no peaks of CHX were detected. The presence of CHX was determined with the following ToF-SIMS analysis for the medicated core PVA-PEG-SiO_2_-1x-CHX and core–shell PVA-PEG-SiO_2_-1x-CHX@PVA-GO. ToF-SIMS detected the site of distribution of CHX loaded in the core PVA-PEG-SiO_2_-1x-CHX. These comparative results presented evidence of the local distribution of CHX in the vicinity of the core due to the observation of pristine and CHX-medicated core–shell fibers with FTIR spectroscopy. A detailed description of the TOF-SIMS analysis is given in the section titled Drug Confirmation, where this method determines the chlorine part of CHX.

#### 2.1.4. Thermal Analysis

**Figure 5:**
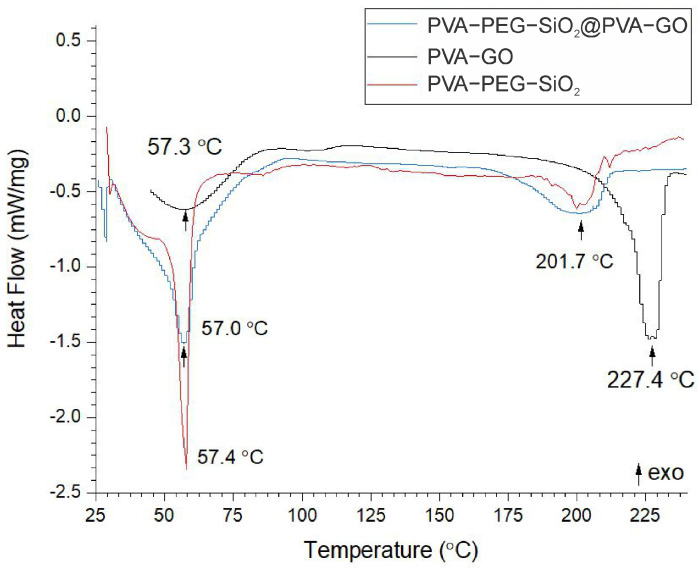
DSC curves of samples with the following compositions: PVA-GO shell; PVA-PEG-SiO_2_ core; PVA-PEG-SiO_2_@PVA-GO core–shell.

Due to the availability of Differential Scanning Calorimetry (DSC) to trace the phase transformation temperatures and the quantification of transformation enthalpies, DSC curves provide information about the phase transition temperature of samples, as given in Figure 5.

DSC measurements of PVA-based fiber mats indicated that the glass transition stage occurred at ca. 57 °C [54]. The PVA-GO fiber mat demonstrated two endothermic peaks located at 57.3 °C and 227.4 °C. The DSC curve of PVA-PEG-SiO_2_ showed the highest peak at 57 °C and a less intensive peak at 201.7 °C. The core–shell fiber mat consisting of the core PVA-PEG-SiO_2_ and shell PVA-GO demonstrates a narrowed peak at 57.4 °C and a broad peak at 201.7 °C. The melting temperature for the core and core–shell compositions seems to be reduced from 227.4 °C to ca. 202 °C due to the amorphous silica and PEG.

The calculated results of the degree of crystallinity for samples and the melting temperature are demonstrated in Table 2.

During the thermal characterization of the shell, core, and core–shell fiber mats, the rise in the melting temperature is noted for the PVA-GO composite. The melting temperature is associated with the coordinated movement of several polymeric molecules, defining the state of structure flexibility [55]. After adding GO, the movement of molecules becomes limited, requiring higher energy input to promote the spatial movement of the monomer chains. This demonstrates the impact of GO on the increase in the crystallinity degree of the polymer composite. In the relevant study, the PCL-GO-based composite showed a higher degree of crystallinity compared to the pristine PCL, which confirmed the efficient function of GO as the modifying agent towards the higher mechanical resistance [56]. GO contributes to the physical properties of a composite, including the crystallinity of the received composite, as in the relevant study [57]. Our results show that the modification of PVA with GO results in an increased degree of crystallinity and melting temperature.

#### 2.1.5. Mechanical Analysis

**Figure A1:**
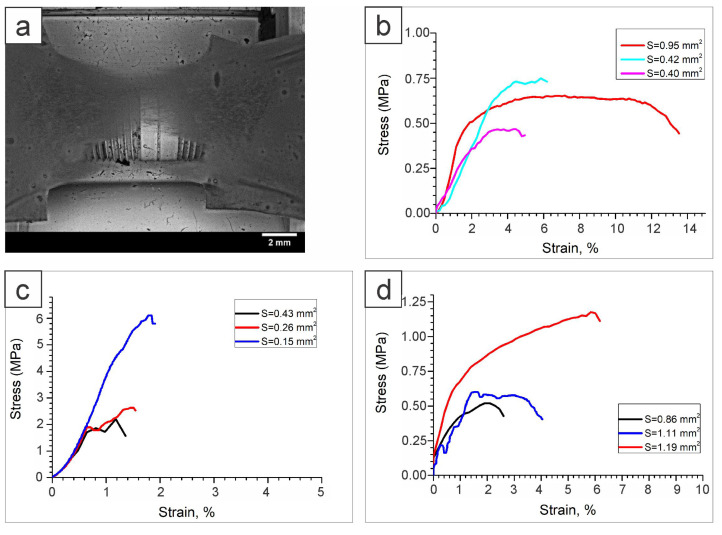
Core–shell fiber mat mounted in the Deben device (**a**); stress–strain curves with the cross-sectional areas of fibers: PVA-GO shell (**b**); PVA-PEG-SiO_2_ core (**c**); PVA-PEG-SiO_2_@PVA-GO core–shell (**d**).

The stress–strain curves of tested samples versus the cross-section areas are given in Appendix A, Figure A1. The calculated tensile strength and elastic modulus of the core, shell, and core–shell fiber mats are included in Table 3.

The tensile test of the core (PVA-PEG-SiO_2_) sample showed the fastest crack propagation among the tested shell and core–shell. The silica nanoparticles as the centers of heterogeneity in the PVA-PEG-SiO_2_ composite cause a faster failure at 1.49 ± 0.25% of elongation. Meanwhile, the PVA-GO-derived shell has an extended period of tensile deformation, where the fracture occurs at 7.07 ± 2.29% of elongation. The core–shell composite demonstrates the breakage of the sample starting at 3.97 ± 1.37% of elongation.

The mechanical performance of the core–shell composite is complex, consequently attributed to the input of the core and shell parts and their bonding. Nanofibers, as an extrinsic toughening approach, provide more extended crack propagation via a bridging effect within the failure of the fiber composite. The tensile resistance of the PVA-GO shell is the highest among the tested samples, with the elongation at break occurring at 7.07 ± 2.29%. Notably, PVA-GO fibers have the smallest diameter, which correlates with the studies reporting that a decreasing fiber diameter is a good fracture-toughening approach via the modification of the plastic deformation mechanism at the crack tip [58,59]. The diameter of fibers could affect the degree of multiple necking of fibers formed during the deformation of the composite.

Core PVA-PEG-SiO_2_ with silica incorporation obtains the highest elastic modulus of 263.17 ± 96.24 MPa with the lowest break at 1.49 ± 0.25% of strain. Meanwhile, the core–shell PVA-PEG-SiO_2_@PVA-GO fibers with the associated GO crosslinkers show the elastic modulus of 35.89 ± 9.70 MPa with the breakage of sample prolonged at 3.97 ± 1.37% of strain. Hence, the increased tensile resistance highlights the synergetic impact of GO in the shell of the core–shell composite. The PVA-GO-derived shell protects the core fiber via hydrogen bonding, with higher tensile resistance in this study and the Refs. [60,61]. The GO-derived shell provides a successful toughening effect, improving the brittle behavior of the core as a part of the core–shell PVA-PEG-SiO_2_@PVA-GO to hinder crack propagation and consequent failure. In our study, the mechanical tests show that the nanomaterials create a well-bonded system that evenly exerts the applied load along the composite.

#### 2.1.6. Biocompatibility Analysis

**Figure 6:**
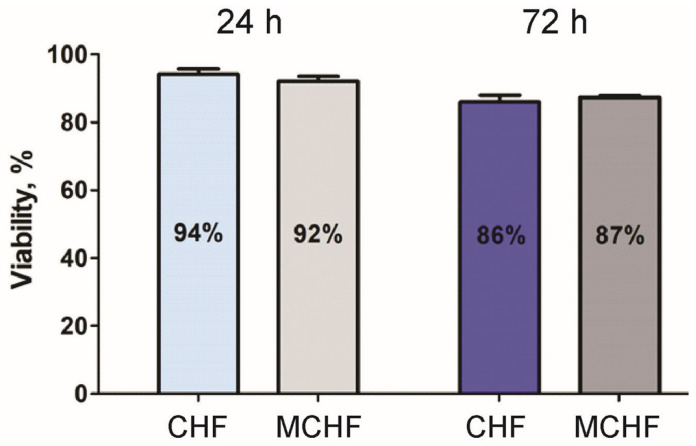
Cell viability assay of PVA-PEG-SiO_2_@PVA-GO core–shell fibers (CHF) and methanol-crosslinked core–shell fiber mats (MCHF).

The cell viability of pristine or ‘empty’ core–shell PVA-PEG-SiO_2_@PVA-GO and core–shell fibers crosslinked with methanol was studied to assess the biocompatibility of the composite. Methanol treatment is one of the primary physical crosslinking methods, based on the formation of hydrogen bonds [30,62]. The effect of GO-based biomaterials on the viability of primary human fibroblast cells was evaluated (Figure 6).

As-prepared and methanol-processed core–shell fibers do not show acute toxicity after 24 h but affect the cell survival or proliferation after 72 h. Considering the biocompatibility, we could conclude that the electrospun core–shell fiber has non-toxic properties in the first 72 h. The results demonstrate that crosslinking with methanol does not significantly affect cell viability compared with the as-prepared fibers. The findings compensate for the lack of cell viability tests in our previous study of physical crosslinking with methanol of core–shell fibers PVA-PEG-SiO_2_@PVA-GO [30]. Here, we report the compatibility of electrospun core–shell fiber composite PVA-PEG-SiO_2_@PVA-GO for human fibroblasts.

### 2.2. Analysis of Fibers Encapsulated with CHX PVA-PEG-SiO_2_-1x-CHX@PVA-GO

#### 2.2.1. Drug Confirmation

Time of flight (ToF)–secondary-ion mass spectrometry (SIMS) has many potential applications and was used to determine the chemical composition of the drug-loaded fibers. This technique was used to trace the CHX-medicated core–shell fiber PVA-PEG-SiO_2_-1x-CHX@PVA-GO. CHX is characterized by several fingerprint peaks at the *m*/*z* of 35, 37, 151, 505 [63].

**Figure 7:**
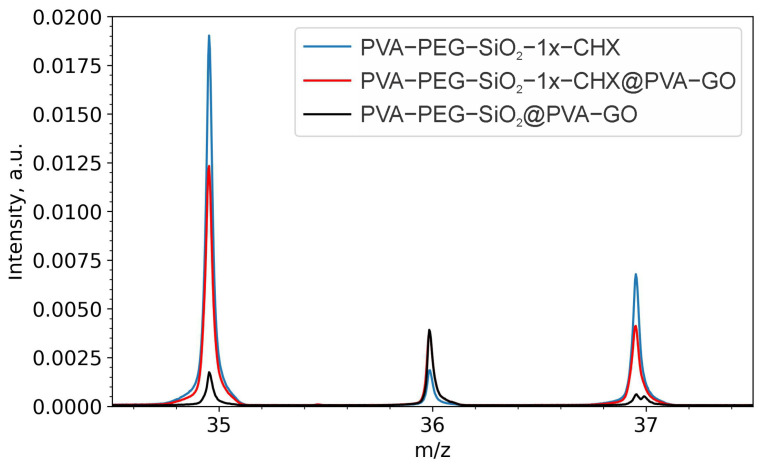
ToF-SIMS spectra obtained in the negative ion mode of core PVA-PEG-SiO_2_-1x-CHX, core–shell PVA-PEG-SiO_2_-1x-CHX@PVA-GO and core–shell PVA-PEG-SiO_2_@PVA-GO.

Regarding the natural isotope distribution pattern of chlorine, the ratio of ^35^Cl/^37^Cl∼3/1 evidences the presence of chlorhexidine. In Figure 7, the chlorine ion intensity (*m*/*z* = 35) is 10.7 times higher for the CHX-loaded core fiber PVA-PEG-SiO_2_-1x-CHX than the ‘empty’ core–shell PVA-PEG-SiO_2_@PVA-GO. Compared with the PVA-PEG-SiO_2_-1x-CHX core fiber, the signal (*m*/*z* = 35) of the core–shell fiber with encapsulated CHX (PVA-PEG-SiO_2_-1x-CHX@PVA-GO) showed a decrease in intensity by 1.6 times. ToF-SIMS is an efficient monitoring tool for encapsulated drugs inside multicomponent core and core–shell fibers.

#### 2.2.2. Drug Encapsulation Efficiency

**Figure 8:**
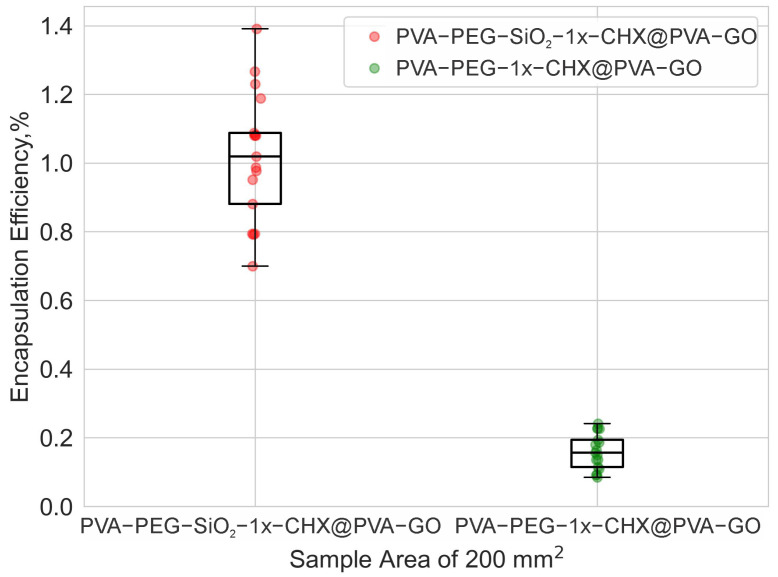
Encapsulation efficiency of drug for PVA-PEG-1x-CHX@PVA-GO, PVA-PEG-SiO_2_-1x-CHX@PVA-GO.

Drug encapsulation was calculated using the optical density of collected absorbance spectra. The silica-based PVA-PEG-SiO_2_-1x-CHX@PVA-GO core–shell showed higher encapsulation efficiency of 1.02 ± 0.27%, where silica nanoparticles captured chlorhexidine more efficiently, providing a greater codelivery function. The core–shell with no silica PVA-PEG-1x-CHX@PVA-GO presented encapsulation efficiency of 0.16 ± 0.05%, as seen in Figure 8.

The incorporation of GO with its potential drug-loading capacity is speculated as a future direction to explore the functionality of coaxial electrospinning [64]. This will be the next task for further investigation.

#### 2.2.3. In Vitro Drug Kinetics

**Figure 9:**
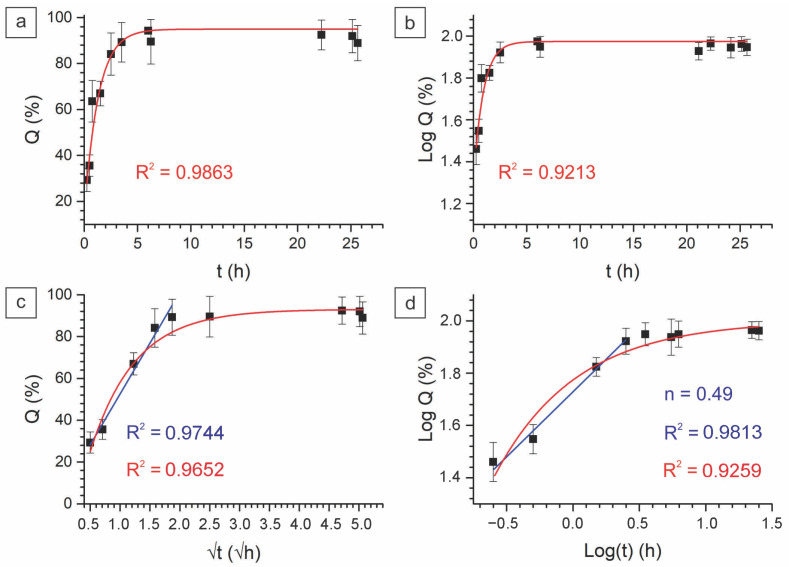
Release profiles of core–shell fiber PVA-PEG-SiO_2_-1x-CHX@PVA-GO fitted to the models: (**a**) zero order, (**b**) first order, (**c**) Higuchi, and (**d**) Korsmeyer–Peppas model.

**Figure 10:**
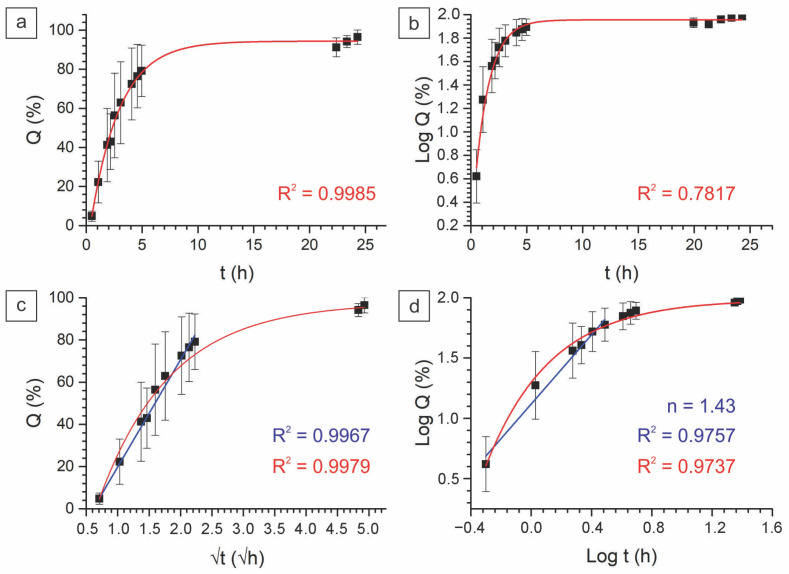
Release profiles of core–shell fiber PVA-PEG-1x-CHX@PVA-GO fitted to the models: (**a**) zero order, (**b**) first order, (**c**) Higuchi, and (**d**) Korsmeyer–Peppas models.

The mechanism of drug release was studied using the zero, first-order, Higuchi, and Korsmeyer–Peppas models. The relevant research reported the Fickian mechanism of drug kinetics for chitosan-crosslinked PVA nanofibers [65]. In our study, the release profiles of the produced fiber samples were better correlated with the Korsmeyer–Peppas and Higuchi models, as in the relevant studies [66,67]. A good correlation with the Higuchi model indicates the case where drug release is provided by diffusion within the pores’ heterogeneous matrix. Fitting to the Korsmeyer–Peppas model gives a slope of curve as a release exponent (*n*) > 0.45, which indicates the non-Fickian diffusion of the drug in the medium. Figure 9 and Figure 10 demonstrate that the release rate of PVA-PEG-SiO_2_-1x-CHX@PVA-GO (*n* = 0.49) is slower than that of PVA-PEG-1x-CHX@PVA-GO (*n* = 1.43). The release exponent value of PVA-PEG-1x-CHX@PVA-GO (*n* = 1.43) indicates the high rate of erosion of the polymer matrix.

The non-Fickian diffusion of silica-containing fibers reveals the case whereby the rate of polymer chain relaxation is significantly slower than the rate of molecule diffusion within the formed gel [66]. The crosslinking mechanism of core–shell nanofiber composite PVA-PEG-SiO_2_@PVA-GO supports the hydrogel’s transformation to hinder the dissociation of the hydrophilic PVA and PEG matrix. This provides the sustainable drug release of PVA-PEG-SiO_2_@PVA-GO compared with the non-silica core–shell composite. Summarizing these findings, we could conclude that the nanosized silica and graphene oxide make significant contributions to the pharmacokinetic properties. During the hydration, the electrospun nanofibers with hydrogen bonding transform in the hydrogel, which highlights the potential for the electrospinning of crosslinked solutions to reinforce the hydrogels for better operability.

#### 2.2.4. Antibacterial Activity

**Figure 11:**
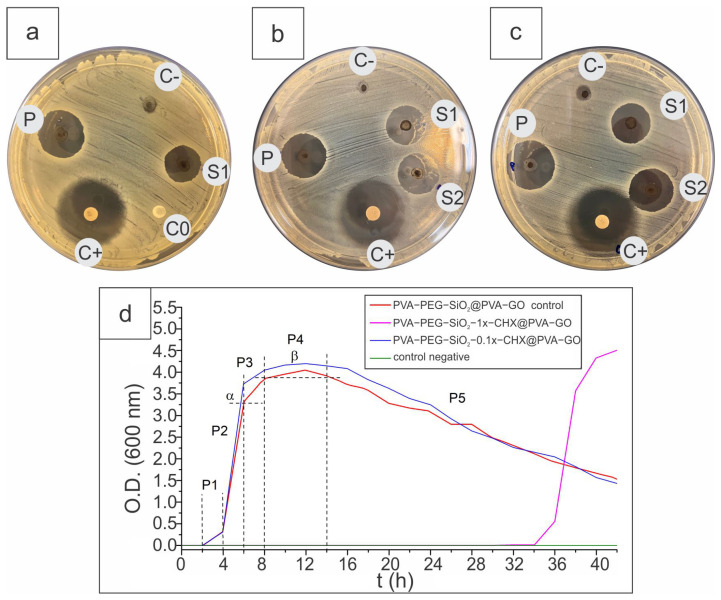
Disc diffusion assays of core–shell fibers (**a**–**c**), where PVA-PEG-SiO_2_@PVA-GO (C-); PVA-PEG-SiO_2_-1x-CHX@PVA-GO (S1, S2), PVA-PEG-1x-CHX@PVA-GO (P), cellulose disk (C0); cellulose control with doxycycline (C+) against *S. aureus*; (**d**) antibacterial activity of CHX-loaded core–shell PVA-PEG-SiO_2_-1x-CHX@PVA-GO and PVA-PEG-SiO_2_-0.1x-CHX@PVA-GO.

The antibacterial activity of the fiber mats was assessed with the disk diffusion method and with optical density measurements. All CHX encapsulated core–shell fiber mats showed antibacterial activity with an obviously traced inhibition halo, as seen in Figure 11a–c. CHX-loaded PVA-PEG-1x-CHX@PVA-GO (P) had an inhibition zone of 71.92 ± 2.48% when the control C+ was assumed as 100%. Samples with silica PVA-PEG-SiO_2_-1x-CHX@PVA-GO (S1, S2) had a smaller inhibition halo, affected by the slower rate of diffusion known from the drug release. For the S1 sample, the inhibition zone reached 58.99 ± 2.03%, while S2 with the fiber synthesis time increased by 1 h had a larger inhibition zone with 65.32 ± 1.66%. The disk diffusion assay revealed the antibacterial efficiency of CHX-medicated core–shell fibers against *Staphylococcus aureus* (*S. aureus*).

The bacterial response was observed, where the effective concentration of CHX (0.1x, 1x) was investigated using the optical density determination of bacterial activity at 600 nm (Figure 11d).

According to the results of cultivation in PVA-PEG-SiO_2_@PVA-GO as a control, the adaptive lag phase lasted for up to 2 h of the experiment. Based on the background of the formation and preparation of enzymatic complexes of bacterial cells, the exponential transition phase was observed from 2 h to 8 h, divided into the following stages: P1—a period of acceleration (2–4 h), P2—a period of intensive logarithmic cell growth (4–6 h), P3—a period of decaying growth (6–8 h).

By the sixth hour of the experiment, a key indicator of optical density (α) was achieved, characterizing the maximum value of colony-forming units in this period ${\mathrm{OD}}_{600nm}$ = 3.25. The period of decaying growth (6–8 h) was marked by a decrease in the speed of generative processes. The second key point was reached with an optical density that corresponded to the M-concentration for microorganisms ${\mathrm{OD}}_{600nm}$ = 3.78 at 8 h. At the interval of 8–14 h, the phase of the stationary position of the cells was observed, without significant fluctuations, with the mean ${\mathrm{OD}}_{600nm}$ = 3.88 in this β segment (8–14 h). After 16 h, there was a phase involving the gradual death of bacterial cells.

Compared with the control (core–shell fiber PVA-PEG-SiO_2_@PVA-GO), the studied sample PVA-PEG-SiO_2_-0.1x-CHX@PVA-GO showed an increase in the α and β indicators by 13% and 5.3%, respectively, without a relapsed lag phase. The cultivation results of the sample PVA-PEG-SiO_2_-1x-CHX@PVA-GO showed an extension of the lag phase up to 30 h, followed by accelerated bacterial growth relative to control values, with the following indicators: the α increased by 30%, and the β increased by 16%. These optical density measurements demonstrate that CHX encapsulation affects the sensitivity of bacterial colonies and slows down their growth for up to 30 h.

The agar disc diffusion assay accompanied by spectrophotometry shows the antibacterial activity of CHX-encapsulated core–shell samples PVA-PEG-SiO_2_-1x-CHX@PVA-GO against the Gram-positive bacterium *S. aureus*. The PVA-PEG-SiO_2_-1x-CHX@PVA-GO sample showed a massive extension of the adaptive lag phase compared with PVA-PEG-SiO_2_-0.1x-CHX@PVA-GO, where the initial concentration of CHX was decreased by 10 times.

The results showed the outcomes of pharmacokinetics as the time-dependent nature of antibacterial efficacy. The next challenging task is to resolve the pharmacodynamic properties of the tested composites as effective drug concentrations. The response regarding the drug dosage should be carefully investigated to be consistent with toxicity in further preclinical studies.

## 3. Materials and Methods

### 3.1. Materials

PVA powder with the molecular weight (MW) 72 kg/mol, 85–89% hydrolyzed, and PEG 8000 BioChemica with MW 7–9 kg/mol were supplied by AppliChem GmbH (Darmstadt, Germany). Phosphate-buffered saline (PBS), fluorescent agents of 5(6)-Carboxyfluo-rescein (FAM), and Rhodamine B (Rh B) were purchased from Sigma-Aldrich (St. Louis, MO, USA). Chlorhexidine digluconate (CHX) was obtained in a pharmaceutical form in Moscow, Russia. Methanol (≥99.5%) was supplied by Chimmed (Moscow, Russia). The ready-to-use suspensions of silicon oxide nanoparticles with a concentration of 0.8 μg/μL and graphene oxide with a concentration of 30 μg/μL were synthesized in Dr. S. Evlashin’s laboratory [52,68]. Dulbecco’s Modified Eagle Medium (DMEM; no. 12491-015), fetal bovine serum (FBS; no. 16000-044), antibiotic–antimycotic (no. 15240-062), and 0.25% trypsin/EDTA (no. 25200-114) were obtained from Gibco, Thermo Fisher Scientific, Waltham, MA, USA. L-glutamine (no. F032) and Versen (no. F080) were received from PanEco, Moscow, Russia. CellTiter-Glo 2.0 (no. G7572) was supplied by Promega, Madison, WI, USA.

### 3.2. Fiber Fabrication

The core–shell fiber was retrieved from core and shell solutions. A mixture of PVA and PEG at 70:30 was dissolved in MilliQ water at 70 °C, 600 rpm in the magnetic stirrer Hei-Standard (d = 145 mm, Heidolph Instruments GmbH & Co. KG, Schwabach, Germany) for 2 h. The polymer ratio of the core solution was 12 wt.%. The ratio of the silica suspension was 7.5 wt.% for core PVA-PEG-SiO_2_ and it was subsequently sonicated via ultrasound for 10 min and stirred for 12 h. The final concentration of silica was 0.049 mg/mL in the core PVA-PEG-SiO_2_ solution.

For the shell PVA-GO, the graphene oxide suspension was mixed with the aqueous polymer solution PVA 10 wt.% dissolved at 80 °C with a rate of 250 rpm for 2 h. The as-received graphene oxide solution was sonicated in the Elmasonic S10H bath (Elma Schmidbauer GmbH, Singen, Germany) for 10 min and added to the PVA solution with the loading of 10 wt.%. The concentration of GO flakes was 1 mg/mL for the PVA-GO-derived shell.

Drug encapsulation was provided with the loading of CHX in the core solution with the fraction of drug 10 mg/mL, 1 mg/mL as PVA-PEG-SiO_2_-1x-CHX and PVA-PEG-SiO_2_-0.1x-CHX, respectively. The received core solutions PVA-PEG-SiO_2_-1x-CHX and PVA-PEG-SiO_2_-0.1x-CHX were used for medicated core and core–shell fibers PVA-PEG-SiO_2_-1x-CHX@PVA-GO and PVA-PEG-SiO_2_-0.1x-CHX@PVA-GO. The solutions were placed in the setup from our previous study [30]. The core and shell solutions were electrospun with a flow rate of 1 mL/h. The fiber synthesis was implemented with a tip-to-target distance of 14 cm, and an operational voltage of 21 kV at room temperature for 3 h. Postprocessing of the core–shell fiber included the immersion of core–shell fibers in 2 mL of methanol for 12 h. Afterward, the methanol-crosslinked fiber mats were collected in a Petri dish and dried at ambient temperature in the hood for 24 h.

### 3.3. Raman and FTIR Spectroscopy

The fibers and as-received GO were monitored with a DXR3xi Raman imaging microscope (Thermo Fisher Scientific, Waltham, MA, USA). Raman spectroscopy was conducted with the laser source at 532 nm, where GO and PVA-GO samples were studied at the laser power of 3 mW and 5 mW, respectively. Core–shell fibers were studied at 6 mW, and all spectra were collected in the range of 200–3200 cm^−1^. The FTIR spectra of PVA-based fiber mats were measured using a Bruker ALPHA II spectrometer (Ettlingen, Germany) in attenuated total reflectance mode with a diamond crystal placed inside a nitrogen-filled glovebox. The spectra were collected in the range of 400–4000 cm^−1^.

### 3.4. DSC

The thermal behavior of core–shell, shell, and core fibers was studied using a differential scanning calorimeter, NETZSCH DSC 204F1 Phoenix (NETZSCH-Gerätebau GmbH, Selb, Germany). DSC thermograms of samples were obtained from 26 °C to 240 °C with a heating rate of 10 °C/min.

DSC thermograms provide information about the melting temperature ($T_{m}$), the heat of melting ($\Delta H_{m}$), and the degree of crystallinity ($\chi_{c}$). The melting temperature was calculated from the DSC curves. The degree of crystallinity was calculated with Formula (1):

(1) $$\chi_{c}\;=\;\frac{\Delta H_{m}\times 100\%}{\Delta H_{c}},$$

where $\Delta H_{m}$ is the heat of melting calculated from the area under the melting peak; $\Delta H_{c}$ is the heat required for the melting of 100% crystalline PVA (138.6 J/g), derived from Refs. [62,69].

### 3.5. Tensile Tests

The prepared specimens were tested with the Deben Microtest 200 N Tensile Stage (Deben UK Ltd., Woolpit, UK) in tension mode with a permanent speed of 1.0 mm/min inside the chamber of a Tescan Vega3 SEM (TESCAN ORSAY HOLDING, Brno, Czech Republic). The double-notched samples were cut from PVA-GO as a shell, PVA-PEG-SiO_2_ as a core, and PVA-PEG-SiO_2_@PVA-GO as the core–shell fiber mat. A sample had a geometry of 25 mm × 14 mm with a length of 2 mm per notch. The thickness of the fiber mats was measured with a micrometer. The tension was parallelly synchronized with SEM image acquisition with the following settings: back-scattered electron (BSE) detector, resolution 1024 × 1024 pixels, 8-bit, scan speed 3.2 μm per pixel, and field of view 1 mm × 1 mm. Then, images were processed via the robust Digital Image Correlation (DIC) method to obtain true material deformations. The DIC technique was realized by open-source Matlab-based software program, Ncorr v.1.2 (GitHub, Inc., San Francisco, CA, USA) [70]. Two characteristics were determined, namely the elastic modulus and ultimate strength.

### 3.6. ToF-SIMS

As-received core–shell (PVA-PEG-SiO_2_@PVA-GO) and CHX-encapsulated core–shell (PVA-PEG-SiO_2_-1x-CHX@PVA-GO) fibers and the core (PVA-PEG-SiO_2_-1x-CHX) were analyzed using the Ga FIB-SEM Tescan S9000 (Tescan Orsay Holding, Brno-Kohoutovice, Czech Republic) equipped with a ToF-SIMS detector (TOFWERK AG, Thun, Switzerland). The following parameters of FIB were used for ‘empty’ core–shell and drug-encapsulated core fibers: high voltage of 20 kV, current of 4 nA, 50 μm field of view, ToF-SIMS: 512 × 512 pixel resolution, negative and positive modes, binning 4 × 4, dwell time 20 μs. The PVA-PEG-SiO_2_-1x-CHX@PVA-GO sample was scanned in the equivalent mode but with a current of 17 nA. ToF-SIMS data were analyzed using the software TOFWERK Explorer 1.12.2.0, Thun, Switzerland.

### 3.7. UV Spectroscopy

The release and encapsulation of CHX were monitored for ten samples of each core–shell fiber composite PVA-PEG-1x-CHX@PVA-GO and PVA-PEG-SiO_2_-1x-CHX@PVA-GO (total 20 samples). Each sample corresponding to 3 mg with 10 mm × 20 mm was attached to an adhesive polyimide film, namely Kapton^®^, fitted into a test tube with 1 mL of 0.01 M PBS and stored in an incubation shaker TS-100 (BioSan, Riga, Latvia) at 37 °C and 100 rpm. These conditions were adopted to mimic a wound dressing on the skin. Aliquot parts of 200 μL were withdrawn from the test tubes and centrifuged to remove GO flakes in a MiniSpin^®^ (Eppendorf AG, Hamburg, Germany) at 10 rpm for 4 min. Then, the spectra were collected in the UV-transparent microplate at 230–1000 nm intervals for the absorbance spectra with the scan step of 2 nm and a number of flashes of 15 in an Infinite M Nano+ (Tecan Trading AG, Männedorf, Switzerland). Subsequently, the supernatant was returned to the dissolution medium in a tube for each release measurement for at least 24 h.

The encapsulation efficiency of CHX was assessed for PVA-PEG-1x-CHX@PVA-GO and PVA-PEG-SiO_2_-1x-CHX@PVA-GO fibers with ten samples for each type. Encapsulation efficiency was calculated by Formula (2), where $m_{d}$ is the total mass of the released drug divided by $m_{d0}$, the total mass of the drug added in the spinning solution.

(2) $$\mathrm{Encapsulation}\;\mathrm{efficiency}=\frac{m_{d}\times 100\%}{m_{d0}}$$

To calculate the concentration of the released drug, the calibration curves were built from the series of UV absorbance spectra for CHX dissolved in PBS with the concentrations (0.004–0.063 mg/mL).

### 3.8. Cell Viability Assay

The experiment was supported by Dr. E. V. Koudan and Dr. F. S. Senatov from the National University of Science and Technology MISIS. Primary human fibroblasts passaged 3 times were cultured in DMEM containing 2 mM L-glutamine and an antimycotic–antibiotic solution (1x), with the addition of 10% (*v*/*v*) fetal bovine serum, at 37 °C and 5% CO_2_. Then, the cells were transferred from the substrate with the use of a Versen solution and a 0.25% trypsin/EDTA solution.

Extracts were prepared by incubating the core–shell samples (PVA-PEG-SiO_2_@PVA-GO, methanol-crosslinked PVA-PEG-SiO_2_@PVA-GO) in a culture medium for 24 h at 37 °C. Each sample had a geometry of 10 mm × 20 mm. The cytotoxicity of core–shell fibers was assessed with the CellTiter-Glo 2.0 system according to the protocol for the biological evaluation of medical devices (GOST ISO-10993). Cells were seeded in a 96-well culture plate at a concentration of 1 × 10^4^ cells per well. Each well contained 100 μL of cell suspension. The plate was incubated for 24 h at 37 °C in a humidified atmosphere with 5% CO_2_ to obtain a monolayer cell culture. After 24 h, 200 μL of extracts was added to each experimental well, and 200 μL of fresh culture medium was added to the control wells. Plates were incubated for 24 h and 72 h at 37 °C in a humidified atmosphere with 5% CO_2_. After 24 h and 72 h, 250 μL of supernatant was withdrawn from each well with the addition of 50μL of CellTilter-Glo 2.0 and incubated for 15 min. Obtained solutions were placed in a 96-well white opaque culture plate to record luminescence signals with the Varioskan LUX Plate Reader (Thermo Fisher Scientific, Vantaa, Finland). Wells containing pure culture medium without cells were used to evaluate the background signal. Absolute luminescence values were normalized as follows: the luminescence signal of cells in control wells (in culture medium) was taken as 100% viability. Cell viability was calculated as a percentage of the luminescence signal received from the experimental and control wells.

### 3.9. Disk Diffusion Assay

The experiment was supported by Dr. M. S. Podporin and Prof. E. V. Ippolitov from Moscow State University of Medicine and Dentistry. The antibacterial activity of the CHX-loaded core–shell fiber mats was assessed for *S. aureus* cultivated on a solid medium agar plate. The working concentration of the suspension of bacteria corresponded to the 0.5 McFarland standard (∼1 × 10${}^{8}$ colony forming units (CFU) mL^−1^). CHX-loaded core–shell fiber mats (PVA-PEG@PVA-GO-1x-CHX and PVA-PEG-SiO_2_-1x-CHX@PVA-GO) and ‘empty’ PVA-PEG-SiO_2_@PVA-GO fibers were sectioned into the disks with a diameter of 6 mm. The fiber samples and a cellulose control with Doxycycline were placed in the agar medium with the seeded colony of *S. aureus*. After static incubation at 37 °C for 1 day, the inhibition zones were photographed and measured to assess the bacterial sensitivity/resistivity parameter.

Optical density measurements of core–shell PVA-PEG-SiO_2_@PVA-GO-0.1x-CHX, PVA-PEG-SiO_2_-1x-CHX@PVA-GO, and medium broth as a negative control were implemented using the RTS-1 Bioreactor (BioSan, Riga, Latvia). Optical density (OD) was measured at 600 nm to examine the response of *S. aureus* bacteria for 42 h. The graph was plotted for the OD at 600 nm versus time for the PVA-PEG-SiO_2_@PVA-GO core–shell with 0.1x and 1x concentrations of CHX.

## 4. Conclusions

In this research, the morphology, chemical structure, and physical properties of synthesized fibers were carefully investigated spectroscopically in terms of the effect of hydrogel-inducing additives on the properties of the suggested wound dressing. Raman analysis showed the reduction trend of GO-derived materials during electrospinning. In vitro drug release indicated non-Fickian diffusion, where the dissociation of the core–shell fiber via hydrogen bonds crosslinked with silica and GO was slowed down. This highlights the importance of crosslinking to control drug release. The polymer–nanomaterial interactions support sustainable drug release via the hydrogel’s transformation from the semicrystalline fiber mat. CHX-encapsulated core–shell fibers provide strong antibacterial effects for *S. aureus*. These results open a pathway to the use of CHX-medicated core–shell fibers against the bacterial biofilms related to the treatment of diabetic wounds and periprosthetic and dental infections, as mentioned among the relevant works [71]. The findings of this work provide a robust platform for the design of electrospun core–shell fibers towards a hydrogel wound dressing to treat bacterial inflammation.

Future directions of fiber hydrogels are based on the versatility of electrospinning, which allows the production of aligned fibers and different topographies of coatings for better adhesion and cell proliferation. Considering wound healing, micro-patternation along with the alignment of fibers is elaborated as an approach to increase cell growth, which could become a problem-solving solution for burns and other chronic wounds and injuries.

## Figures and Tables

**Table 1:** FTIR bands attributed to PVA and PEG.

Wavenumber, cm^−1^	Band	Reference
3313	–OH stretching (PVA)	[41]
2933, 2910	CH_2_ stretching (PVA)	[43]
1735	C=O stretching of acetate group (PVA)	[47]
1432	CH_2_ bending (PVA)	[43]
1376	(CH–OH) bending (PVA)	[41]
1242	CH wagging (PVA)	[48]
1145	C–O and C–C stretching bonds (PVA)	[46]
1090	C–O stretching of an acetyl group (PVA)	[44]
942	CH_2_ rocking (PVA)	[48]
845	CC stretching (PVA)	[43]
1343	CH_3_ bending (PEG)	[32]

**Table 2:** Degree of crystallinity and melting temperature of core–shell (PVA-PEG-SiO_2_@PVA-GO), core (PVA-PEG-SiO_2_), and shell (PVA-GO) fibers.

Name	T_*m*_, °C	T_*g*_, °C	Heat of Melting ($\Delta H_{m}$), J/g	Degree of Crystallinity, %
Shell	227.4	57.3	83.02	59.90
Core	201.7	57.0	24.49	17.67
Core–shell	201.7	57.4	48.18	33.32

**Table 3:** The tensile properties of core–shell (PVA-PEG-SiO_2_@PVA-GO), core (PVA-PEG-SiO_2_), and shell (PVA-GO) fibers.

Name	Elstic Modulus, MPa	Strength, MPa	Elongation at Break, %	Mean Diameter, nm
Core	263.17 ± 96.24	3.65 ± 1.75	1.49 ± 0.25	381 ± 131
Shell	19.91 ± 1.67	0.62 ± 0.10	7.07 ± 2.29	174 ± 31
Core–shell	35.89 ± 9.70	0.77 ± 0.29	3.97 ± 1.37	261 ± 51

## Data Availability

Not applicable.

## References

[1] Thomas R.E., Thomas B.C. (2021). Reducing Biofilm Infections in Burn Patients’ Wounds and Biofilms on Surfaces in Hospitals, Medical Facilities and Medical Equipment to Improve Burn Care: A Systematic Review. Int. J. Environ. Res. Public Health.

[2] Chen H., Cheng R., Zhao X., Zhang Y., Tam A., Yan Y., Shen H., Zhang Y.S., Qi J., Feng Y. (2019). An Injectable self-Healing Coordinative Hydrogel with Antibacterial and Angiogenic Properties for Diabetic Skin Wound Repair. NPG Asia Mater..

[3] Lipsky B.A., Senneville É., Abbas Z.G., Aragón-Sánchez J., Diggle M., Embil J.M., Kono S., Lavery L.A., Malone M., van Asten S.A. (2020). Guidelines on the Diagnosis and Treatment of Foot Infection in Persons with Diabetes (IWGDF 2019 Update). Diabetes/Metab. Res. Rev..

[4] Jang C.H., Cho Y.B., Jang Y.S., Kim M.S., Kim G.H. (2015). Antibacterial Effect of Electrospun Polycaprolactone/Polyethylene Oxide/Vancomycin Nanofiber Mat for Prevention of Periprosthetic Infection and Biofilm Formation. Int. J. Pediatr. Otorhinolaryngol..

[5] Teixeira M.O., Antunes J.C., Felgueiras H.P. (2021). Recent Advances in Fiber–Hydrogel Composites for Wound Healing and Drug Delivery Systems. Antibiotics.

[6] Li Y., Wang J., Wang Y., Cui W. (2021). Advanced Electrospun Hydrogel Fibers for Wound Healing. Compos. Part B Eng..

[7] Razali N.A.M., Lin W.C. (2022). Accelerating the Excisional Wound Closure by Using the Patterned Microstructural Nanofibrous Mats/Gentamicin-loaded Hydrogel Composite Scaffold. Mater. Today Bio.

[8] Ahmadian Z., Correia A., Hasany M., Figueiredo P., Dobakhti F., Eskandari M.R., Hosseini S.H., Abiri R., Khorshid S., Hirvonen J. (2021). A Hydrogen-bonded Extracellular Matrix-mimicking Bactericidal Hydrogel with Radical Scavenging and Hemostatic Function for pH-Responsive Wound Healing Acceleration. Adv. Healthc. Mater..

[9] Liang Y., He J., Guo B. (2021). Functional Hydrogels as Wound Dressing to Enhance Wound Healing. ACS Nano.

[10] Mirzaeei S., Taghe S., Asare-Addo K., Nokhodchi A. (2021). Polyvinyl Alcohol/Chitosan Single-Layered and Polyvinyl Alcohol/Chitosan/Eudragit RL100 Multi-layered Electrospun Nanofibers as an Ocular Matrix for the Controlled Release of Ofloxacin: An In Vitro and In Vivo Evaluation. AAPS PharmSciTech.

[11] Kimmins S.D., Hanay S.B., Murphy R., O’Dwyer J., Ramalho J., Ryan E.J., Kearney C.J., O’Brien F.J., Cryan S.A., Fitzgerald-Hughes D. (2021). Antimicrobial and Degradable Triazolinedione (TAD) Crosslinked Polypeptide Hydrogels. J. Mater. Chem. B.

[12] Ahmed A.S., Mandal U.K., Taher M., Susanti D., Jaffri J.M. (2018). PVA-PEG Physically Cross-linked Hydrogel Film as a Wound Dressing: Experimental Design and Optimization. Pharm. Dev. Technol..

[13] Uchino Y., Shimmura S., Miyashita H., Taguchi T., Kobayashi H., Shimazaki J., Tanaka J., Tsubota K. (2007). Amniotic Membrane Immobilized Poly (vinyl alcohol) Hybrid Polymer as an Artificial Cornea Scaffold that Supports a Stratified and Differentiated Corneal Epithelium. J. Biomed. Mater. Res. Part B Appl. Biomater. Off. J. Soc. Biomater. Jpn. Soc. Biomater. Aust. Soc. Biomater. Korean Soc. Biomater..

[14] Hu Y., Yan P., Fu L., Zheng Y., Kong W., Wu H., Yu X. (2017). Polyvinyl Alcohol/Polyvinyl Pyrrolidone Crosslinked Hydrogels Induce Wound Healing through Cell Proliferation and Migration. J. Biomater. Tissue Eng..

[15] Horiguchi S., Adachi T., Rondinella A., Boschetto F., Marin E., Zhu W., Tahara Y., Yamamoto T., Kanamura N., Akiyoshi K. (2019). Osteogenic Response of Mesenchymal Progenitor Cells to Natural Polysaccharide Nanogel and Atelocollagen Scaffolds: A Spectroscopic Study. Mater. Sci. Eng. C.

[16] Qi X., Huang Y., You S., Xiang Y., Cai E., Mao R., Pan W., Tong X., Dong W., Ye F. (2022). Engineering Robust Ag-Decorated Polydopamine Nano-Photothermal Platforms to Combat Bacterial Infection and Prompt Wound Healing. Adv. Sci..

[17] Wang M., Hou J., Yu D.G., Li S., Zhu J., Chen Z. (2020). Electrospun Tri-layer Nanodepots for Sustained Release of Acyclovir. J. Alloys Compd..

[18] Elyaderani A.K., Lama-Odría D., del Carmen M., Valle L.J.d., Puiggalí J. (2022). Multifunctional Scaffolds Based on Emulsion and Coaxial Electrospinning Incorporation of Hydroxyapatite for Bone Tissue Regeneration. Int. J. Mol. Sci..

[19] Darbasizadeh B., Fatahi Y., Feyzi-Barnaji B., Arabi M., Motasadizadeh H., Farhadnejad H., Moraffah F., Rabiee N. (2019). Crosslinked-Polyvinyl Alcohol-Carboxymethyl Cellulose/ZnO Nanocomposite Fibrous Mats Containing Erythromycin (PVA-CMC/ZnO-EM): Fabrication, Characterization and In-Vitro Release and Anti-Bacterial Properties. Int. J. Biol. Macromol..

[20] Fan Z., Guan J. (2016). Antifibrotic Therapies to Control Cardiac Fibrosis. Biomater. Res..

[21] Morimune S., Kotera M., Nishino T., Goto K., Hata K. (2011). Poly (vinyl Alcohol) Nanocomposites with Nanodiamond. Macromolecules.

[22] Jia R.J., Teng K.Y., Huang J.Y., Wei X., Qin Z.Y. (2022). Hydrogen Bonding Crosslinking of Starch-Polyvinyl Alcohol Films Reinforced by Ultrasound-Assisted and Cellulose Nanofibers Dispersed Cellulose Nanocrystals. Starch-Stärke.

[23] Zhang P., Guo W., Guo Z.H., Ma Y., Gao L., Cong Z., Zhao X.J., Qiao L., Pu X., Wang Z.L. (2021). Dynamically Crosslinked Dry Ion-Conducting Elastomers for Soft Iontronics. Adv. Mater..

[24] Dong P., Cui K., Xu F., Jiang T., Ma Z. (2018). Synthesis of New Ionic Crosslinked Polymer Hydrogel Combining Polystyrene and Poly (4-vinyl Pyridine) and Its Self-Healing through a Reshuffling Reaction of the Trithiocarbonate Moiety under Irradiation of Ultraviolet Light. Polym. Int..

[25] Cheng S., Wang H., Pan X., Zhang C., Zhang K., Chen Z., Dong W., Xie A., Qi X. (2022). Dendritic Hydrogels with Robust Inherent Antibacterial Properties for Promoting Bacteria-infected Wound Healing. ACS Appl. Mater. Interfaces.

[26] Bardonová L., Mamulová Kutláková K., Kotzianová A., Kulhánek J., Zidek O., Velebný V., Tokarský J. (2020). Electrospinning of Fibrous Layers Containing an Antibacterial Chlorhexidine/Kaolinite Composite. ACS Appl. Bio Mater..

[27] Massarelli E., Silva D., Pimenta A., Fernandes A., Mata J., Armês H., Salema-Oom M., Saramago B., Serro A. (2021). Polyvinyl Alcohol/Chitosan Wound Dressings Loaded with Antiseptics. Int. J. Pharm..

[28] Pilloni A., Ceccarelli S., Bosco D., Gerini G., Marchese C., Marini L., Rojas M.A. (2021). Effect of Chlorhexidine Digluconate in Early Wound Healing of Human Gingival Tissues. A Histological, Immunohistochemical and Biomolecular Analysis. Antibiotics.

[29] Abdel-Sayed P., Tornay D., Hirt-Burri N., de Buys Roessingh A., Raffoul W., Applegate L.A. (2020). Implications of Chlorhexidine Use in Burn Units for Wound Healing. Burns.

[30] Kan Y., Bondareva J.V., Statnik E.S., Cvjetinovic J., Lipovskikh S., Abdurashitov A.S., Kirsanova M.A., Sukhorukhov G.B., Evlashin S.A., Salimon A.I. (2022). Effect of Graphene Oxide and Nanosilica Modifications on Electrospun Core-Shell PVA–PEG–SiO_2_@ PVA–GO Fiber Mats. Nanomaterials.

[31] James P., Worthington H.V., Parnell C., Harding M., Lamont T., Cheung A., Whelton H., Riley P. (2017). Chlorhexidine Mouthrinse as an Adjunctive Treatment for Gingival Health. Cochrane Database Syst. Rev..

[32] Repanas A., Wolkers W., Gryshkov O., Müller M., Glasmacher B. (2015). PCL/PEG Electrospun Fibers as Drug Carriers for the Controlled Delivery of Dipyridamole. Silico In Vitro Pharm..

[33] Zubair N.A., Rahman N.A., Lim H.N., Zawawi R.M., Sulaiman Y. (2016). Electrochemical Properties of PVA–GO/PEDOT Nanofibers Prepared Using Electrospinning and Electropolymerization Techniques. RSC Adv..

[34] Sagitova E., Prokhorov K., Nikolaeva G.Y., Baimova A., Pashinin P., Yarysheva A.Y., Mendeleev D. (2018). Raman Analysis of Polyethylene Glycols and Polyethylene Oxides. Proc. J. Phys. Conf. Ser..

[35] Pawar P.B., Shukla S., Saxena S. (2016). Graphene Oxide–Polyvinyl Alcohol Nanocomposite Based Electrode Material for Dupercapacitors. J. Power Sour..

[36] Somesh T., Demappa T. (2022). Tailoring of Ternary Nanocomposite Films of Poly (vinyl Alcohol)/AgAlO_2_@ Reduced Graphene Oxide: An Active Material for Flexible Supercapacitors. J. Solid State Chem..

[37] Wadhwa H., Kandhol G., Deshpande U.P., Mahendia S., Kumar S. (2020). Thermal Stability and Dielectric Relaxation Behavior of In Situ Prepared Poly (vinyl Alcohol)(PVA)-reduced Graphene Oxide (RGO) Composites. Colloid Polym. Sci..

[38] Kim S.G., Park O.K., Lee J.H., Ku B.C. (2013). Layer-by-layer Assembled Graphene Oxide Films and Barrier Properties of Thermally Reduced Graphene Oxide Membranes. Carbon Lett..

[39] Khabibullin A., Alizadehgiashi M., Khuu N., Prince E., Tebbe M., Kumacheva E. (2017). Injectable Shear-Thinning Fluorescent Hydrogel Formed by Cellulose Nanocrystals and Graphene Quantum Dots. Langmuir.

[40] Zhang J., Zhang Q., Liu X., Xia S., Gao Y., Gao G. (2022). Flexible and Wearable Strain Sensors Based on Conductive Hydrogels. J. Polym. Sci..

[41] Zidan H.M., Abdelrazek E.M., Abdelghany A.M., Tarabiah A.E. (2019). Characterization and Some Physical Studies of PVA/PVP Filled with MWCNTs. J. Mater. Res. Technol..

[42] Kharazmi A., Faraji N., Hussin R.M., Saion E., Yunus W.M.M., Behzad K. (2015). Structural, Optical, Opto-thermal and Thermal Properties of ZnS–PVA Nanofluids Synthesized through a Radiolytic Approach. Beilstein J. Nanotechnol..

[43] Sanchez Ramirez D.O., Cruz-Maya I., Vineis C., Tonetti C., Varesano A., Guarino V. (2021). Design of Asymmetric Nanofibers-Membranes Based on Polyvinyl Alcohol and Wool-Keratin for Wound Healing Applications. J. Funct. Biomater..

[44] Tretinnikov O., Zagorskaya S. (2012). Determination of the Degree of Crystallinity of Poly (vinyl Alcohol) by FTIR Spectroscopy. J. Appl. Spectrosc..

[45] Halima N.B. (2016). Poly (vinyl Alcohol): Review of its Promising Applications and Insights into Biodegradation. RSC Adv..

[46] Jessie Lue S., Chen J.Y., Ming Yang J. (2007). Crystallinity and Stability of Poly (vinyl Alcohol)-Fumed Silica Mixed Matrix Membranes. J. Macromol. Sci. Part B.

[47] Abdelaziz M., Abdelrazek E. (2007). Effect of Dopant Mixture on Structural, Optical and Electron Spin Resonance Properties of Polyvinyl Alcohol. Phys. B Condens. Matter.

[48] Alghunaim N.S. (2016). Optimization and Spectroscopic Studies on Carbon Nanotubes/PVA Nanocomposites. Results Phys..

[49] Budi H.S., Ansari M.J., Jasim S.A., Abdelbasset W.K., Bokov D., Mustafa Y.F., Najm M.A., Kazemnejadi M. (2022). Preparation of Antibacterial Gel/PCL Nanofibers Reinforced by Dicalcium Phosphate-modified Graphene Oxide with Control Release of Clindamycin for Possible Application in Bone Tissue Engineering. Inorg. Chem. Commun..

[50] Luo Q., Shan Y., Zuo X., Liu J. (2018). Anisotropic Tough Poly (vinyl Alcohol)/Graphene Oxide Nanocomposite Hydrogels for Potential Biomedical Applications. RSC Adv..

[51] Zhang Z., Wu Y., Wang Z., Zhang X., Zhao Y., Sun L. (2017). Electrospinning of Ag Nanowires/Polyvinyl Alcohol Hybrid Nanofibers for their Antibacterial Properties. Mater. Sci. Eng. C.

[52] Bondareva J.V., Aslyamov T.F., Kvashnin A.G., Dyakonov P.V., Kuzminova Y.O., Mankelevich Y.A., Voronina E.N., Dagesyan S.A., Egorov A.V., Khmelnitsky R.A. (2020). Environmentally Friendly Method of Silicon Recycling: Synthesis of Silica Nanoparticles in an Aqueous Solution. ACS Sustain. Chem. Eng..

[53] Feifel S.C., Lisdat F. (2011). Silica Nanoparticles for the Layer-by-Layer Assembly of Fully Electro-active Cytochrome c Multilayers. J. Nanobiotechnol..

[54] Alharbi H.F., Luqman M., Fouad H., Khalil K.A., Alharthi N.H. (2018). Viscoelastic Behavior of Core-shell Structured Nanofibers of PLA and PVA Produced by Coaxial Electrospinning. Polym. Test..

[55] Flores-Arriaga J.C., Chavarría-Bolaños D., Pozos-Guillén A.d.J., Escobar-Barrios V.A., Cerda-Cristerna B.I. (2021). Synthesis of a PVA Drug Delivery System for Controlled Release of a Tramadol–Dexketoprofen Combination. J. Mater. Sci. Mater. Med..

[56] Kołodziej A., Długoń E., Świętek M., Ziąbka M., Dawiec E., Gubernat M., Michalec M., Wesełucha-Birczyńska A. (2021). A Raman Spectroscopic Analysis of Polymer Membranes with Graphene Oxide and Reduced Graphene Oxide. J. Compos. Sci..

[57] Kovaleva P.A., Pariy I.O., Chernozem R.V., Zadorozhnyy M.Y., Permyakova E.S., Kolesnikov E.A., Surmeneva M.A., Surmenev R.A., Senatov F.S. (2022). Shape Memory Effect in Hybrid Polylactide-based Polymer Scaffolds Functionalized with Reduced Graphene Oxide for Tissue Engineering. Eur. Polym. J..

[58] Chew S.Y., Hufnagel T.C., Lim C.T., Leong K.W. (2006). Mechanical Properties of Single Electrospun Drug-Encapsulated Nanofibres. Nanotechnology.

[59] Daelemans L., Verschatse O., Heirman L., Van Paepegem W., De Clerck K. (2021). Toughening Mechanisms Responsible for Excellent Crack Resistance in Thermoplastic Nanofiber Reinforced Epoxies Through In-Situ Optical and Scanning Electron Microscopy. Compos. Sci. Technol..

[60] Fu Z.Z., Guo S.J., Li C.X., Wang K., Zhang Q., Fu Q. (2022). Hydrogen-bond-dominated Mechanical Stretchability in PVA Films: From Phenomenological to Numerical Insights. Phys. Chem. Chem. Phys..

[61] Duan G., Jin M., Wang F., Greiner A., Agarwal S., Jiang S. (2021). Core Effect on Mechanical Properties of One Dimensional Electrospun Core-Sheath Composite Fibers. Compos. Commun..

[62] Miraftab M., Saifullah A.N., Çay A. (2015). Physical Stabilisation of Electrospun Poly (vinyl Alcohol) Nanofibres: Comparative Study on Methanol and Heat-based Crosslinking. J. Mater. Sci..

[63] Judd A.M., Scurr D.J., Heylings J.R., Wan K.W., Moss G.P. (2013). Distribution and Visualisation of Chlorhexidine within the Skin Using ToF-SIMS: A Potential Platform for the Design of More Efficacious Skin Antiseptic Formulations. Pharm. Res..

[64] Quagliarini E., Di Santo R., Pozzi D., Tentori P., Cardarelli F., Caracciolo G. (2020). Mechanistic Insights into the Release of Doxorubicin from Graphene Oxide in Cancer Cells. Nanomaterials.

[65] Cui Z., Zheng Z., Lin L., Si J., Wang Q., Peng X., Chen W. (2018). Electrospinning and Crosslinking of Polyvinyl Alcohol/Chitosan Composite Nanofiber for Transdermal Drug Delivery. Adv. Polym. Technol..

[66] Barani H., Khorashadizadeh M., Haseloer A., Klein A. (2020). Characterization and Release Behavior of a Thiosemicarbazone from Electrospun Polyvinyl Alcohol Core–shell Nanofibers. Polymers.

[67] Mirzaie Z., Reisi-Vanani A., Barati M., Atyabi S.M. (2021). The Drug Release Kinetics and Anticancer Activity of the GO/PVA-curcumin Nanostructures: The Effects of the Preparation Method and the GO Amount. J. Pharm. Sci..

[68] Evlashin S.A., Svyakhovskiy S.E., Fedorov F.S., Mankelevich Y.A., Dyakonov P.V., Minaev N.V., Dagesyan S.A., Maslakov K.I., Khmelnitsky R.A., Suetin N.V. (2018). Ambient Condition Production of High Quality Reduced Graphene Oxide. Adv. Mater. Interfaces.

[69] Guan Y., Li W., Zhang Y., Shi Z., Tan J., Wang F., Wang Y. (2017). Aramid Nanofibers and Poly (vinyl Alcohol) Nanocomposites for Ideal Combination of Strength and Toughness via Hydrogen Bonding Interactions. Compos. Sci. Technol..

[70] Blaber J., Adair B., Antoniou A. (2015). Ncorr: Open-source 2D Digital Image Correlation Matlab Software. Exp. Mech..

[71] Pan W., Qi X., Xiang Y., You S., Cai E., Gao T., Tong X., Hu R., Shen J., Deng H. (2022). Facile Formation of Injectable Quaternized Chitosan/Tannic Acid Hydrogels with Antibacterial and ROS Scavenging Capabilities for Diabetic Wound Healing. Int. J. Biol. Macromol..

